# Characteristics and patient pathways of Lyme disease patients: a retrospective analysis of hospital episode data in England and Wales (1998–2015)

**DOI:** 10.1186/s12889-019-7245-8

**Published:** 2019-08-15

**Authors:** John S. P. Tulloch, Valerie Decraene, Rob M. Christley, Alan D. Radford, Jenny C. Warner, Roberto Vivancos

**Affiliations:** 1 NIHR Health Protection Research Unit in Emerging and Zoonotic Infections, University of Liverpool, Liverpool, L69 3GL UK; 2 Public Health England, Liverpool, L3 1DS UK; 3 Institute of Infection and Global Health, University of Liverpool, Liverpool, CH64 7TE UK; 4 NIHR Health Protection Research Unit in Emerging and Zoonotic Infections, Public Health England, Porton Down, SP4 0JQ UK; 5 Rare and Imported Pathogens Laboratory, Public Health England, Porton Down, SP4 0JQ UK; 6 NIHR Health Protection Research Unit in Emerging and Zoonotic Infections, Public Health England, Liverpool, L3 1DS UK

**Keywords:** Lyme disease, Incidence, Care pathway, Hospital episodes, Secondary care, Hospital care, England, Wales

## Abstract

**Background:**

Lyme disease is a tick-borne disease of increasing global importance. There is scant information on Lyme disease patient demographics in England and Wales, and how they interact with the National Health Service (NHS). Our aims were to explore the demographic characteristics of Lyme disease patients within the Hospital Episode Statistics (HES) and Patient Episode Database for Wales (PEDW), and to describe patient pathways.

**Methods:**

Data from 1st January 1998 to 31st December 2015 was retrieved from the two administrative hospital datasets (HES and PEDW), based on patients coded with Lyme disease. Information was collected on demographic characteristics, home address and case management. Incidence rates were calculated, and demographics compared to the national population.

**Results:**

Within HES and PEDW, 2361 patients were coded with Lyme disease. There was a significant increase (*p* < 0.01) in incidence from 0.08 cases/100,000 in 1998, to 0.53 cases/100,000 in 2015. There was a bimodal age distribution, patients were predominantly female, white and from areas of low deprivation. New cases peaked annually in August, with higher incidence rates in southern central and western England. Within hospital admission data (*n* = 2066), most cases were either referred from primary care (28.8%, *n* = 596) or admitted via accident and emergency (A&E) (29.5%, *n* = 610). This population entering secondary care through A&E suggest a poor understanding of the recommended care pathways for symptoms related to Lyme disease by the general population.

**Conclusions:**

These data can be used to inform future investigations into Lyme disease burden, and patient management within the NHS. They provide demographic information for clinicians to target public health messaging or interventions.

## Background

Lyme disease is an important emerging tick-borne disease caused by members of the spirochaetal complex *Borrelia burgdorferi* sensu lato. The population-weighted incidence across Western Europe has been estimated to be 22.04/100,000 person-years [1]. In England and Wales the national incidence of laboratory confirmed cases has risen from 0.38 per 100,000 population in 1997 [2] to 1.95 per 100,000 population in 2016 [3, 4]. Lyme disease is associated with a range of clinical presentations which may vary as infection progresses, though it commonly presents as erythema migrans with associated flu-like symptoms [5]. Other presentations include; borrelial lymphocytoma, Lyme neuroborreliosis, carditis, arthritis and acrodermatitis chronica atrophicans (ACA) [6]. This has resulted in broad and varied case definitions [5, 7–9]. However, UK and European case definitions agree that erythema migrans alone, without any laboratory confirmation, is sufficient for case confirmation [5, 7]. Considering this, current surveillance for England and Wales, which is based on laboratory diagnosis [2], is likely to underestimate the true incidence of disease [4]. This resulted in the recent NICE (National Institute for Health and Care Excellence) guidelines explicitly stating that ‘there is a lack of robust epidemiological data on Lyme disease in the UK’ [5].

Patients may present with Lyme disease in either a primary care or hospital setting, with an unknown proportion receiving confirmatory laboratory diagnosis. The relative proportion of patients presenting to either setting is currently unknown, as is the patient pathway between primary, secondary and tertiary care. Within England and Wales, studies that describe patients in a hospital setting have been either limited to one hospital [10, 11], specialist referral centres [12–14], or one clinical presentation [15].

Since 1989 Hospital Episode Statistics (HES) have recorded every ‘episode’ of admitted patient care (APC) delivered in National Health Service (NHS) hospitals in England [16]. Outpatient attendance (OA) and accident and emergency departments (A&E) datasets were added in 2003 and 2007. Patient Episode Database for Wales (PEDW) is a central administrative database that collects admissions data from NHS hospitals in Wales [17, 18]. The primary use of these data is the calculation of health care costs and therefore mainly administrative data is collated. There is now an increasing body of medical research using the HES and PEDW databases; nevertheless, a recent systematic review highlighted that only 17 out of 148 HES publications were related to the epidemiology of a specific disease [19].

The aim of this study was to perform a retrospective analysis of HES and PEDW records to describe the incidence and demographics of Lyme disease patients in a hospital setting, and to describe their patient pathways through the NHS.

## Methods

We performed a retrospective search of both HES (including all datasets of APC, OA and A&E) and PEDW databases to identify patients coded with Lyme disease. A case was defined as a patient with a Lyme disease diagnostic code drawn from the International Statistical Classification of Diseases and Related Health Problems 10th Revision (ICD-10) (Table 1) [5, 7–9, 20].

**Table 1:** Lyme disease ICD-10 codes used to query hospital administrative data

ICD-10 Code	Description
A69.2	Lyme disease
M01.2	Arthritis in Lyme disease
L90.4	Acrodermatitis chronica atrophicans

ICD-10 codes have been used in NHS hospitals since 1995. Therefore, all the hospitals in this study used this coding system throughout the study period. A list of variables for each dataset within HES and PEDW was constructed. These variables could be split into three categories; patient demographics, patient geography, and patient management (Table 2).

**Table 2:** Variables queried of Lyme disease coded patients in hospital administrative data

Hospital Episode Statistic variable codes	Patient Episode Database for Wales variable codes	Description
HESID	Patient ID	Unique pseudoanonymised patient identifier
ADMIAGE	Admitted Age	Age on day of admission
ADMIDATE	Date first admitted	Date of admission
ADMISOURCE	Admission Method	Source of admission
AEARRIVALMODE		Accident and emergency source
AEATTENDDISP		Accident and emergency discharge destination
APPTAGE		Age on day of appointment
APPTDATE		Appointment date
ARRIVALAGE		Age on arrival to accident and emergency
ARRIVALDATE		Date on arrival to accident and emergency
ATTENDED		Did or did not attend outpatient appointment
DEPDUR		Time spent in accident and emergency until departure
DIAG_CODE		Diagnose code searched in all diagnosis code fields
DISDEST		Discharge destination
EPIDUR		Duration of episode
ETHNOS		Ethnicity
IMD04		Index of Multiple Deprivation
	Deprivation Index	Welsh Index of Multiple Deprivation
LSOA11	LSOA_Code	Lower super output area – 2011 census
REFSOURCE		Source of referral for outpatients
RURURB_IND	Urban Indicator	Rural-urban indicator
SEX	Sex	Sex
TRETSPEF		Main treatment speciality

Data were extracted for patients presenting between 1 January 1998 and 31 December 2015 who had a Lyme disease code in any of the diagnostic fields. Data was cleaned by identifying missing values and de-duplication of records. Date of first appearance of a patient within any of the databases, based on pseudo-anonymised patient identifiers and admission date, was used for analysis. Using these index records, the incidence of Lyme disease coded patients was described for each dataset; mid-year population estimates provided by the Office for National Statistics (ONS) were used as the denominator population data [21]. Annual incidences were analysed using linear regression.

Information on patient sex was stratified by age and compared using a binomial test. Ethnicity was compared to national figures available from the ONS using a Chi-squared test [21]. The average annual incidence was calculated at the geographical area of local authority. The rural-urban indicators of the study populations were compared to the national population using a Chi-squared test.

Associations were assessed using linear regression for the Index of Multiple Deprivation (IMD) of English patients, whereas the Welsh Index of Multiple Deprivation (WIMD) of Welsh patients was assessed using a Chi-squared test for trend. Linear regression could not be performed on WIMD as the defined WIMD groups were of uneven proportion, unlike the IMD which is organised in equally sized deciles. Both were compared to the national populations using a Chi-squared test of independence.

Information relating to patient management primarily was analysed descriptively. To determine if any ‘day of the week’ bias existed in the data, the number of cases per day was compared to the expected number of cases per day, using a Chi-squared test. This was performed for each dataset and by the admission method recorded in the APC dataset, with the null hypothesis being that there were an equal number of cases every day of the week.

Pseudoanonymised patient identifiers were used to describe the patient pathway. Statistical analyses were carried out using R (version 3.2.0) (R Core Team 2015), and associations were deemed significant where a *p* value was less than 0.05.

## Results

After de-duplication, 2361 patients were identified with Lyme disease codes between 1998 and 2015. Within English records (HES) 2259 unique patients were identified, 2045 of these were found in APC alone, 180 in outpatients, 13 in A&E, 18 were found in APC and outpatients, and three were found in APC and A&E. Within Welsh records (PEDW), 102 patients were identified. Even though they could not be linked with the HES databases, these were likely to be unique patients, as none of them shared age, sex and lower super output area (LSOA) of home address combinations with any HES patients. We therefore described the combined results of both datasets unless otherwise specified.

**Fig. 1:**
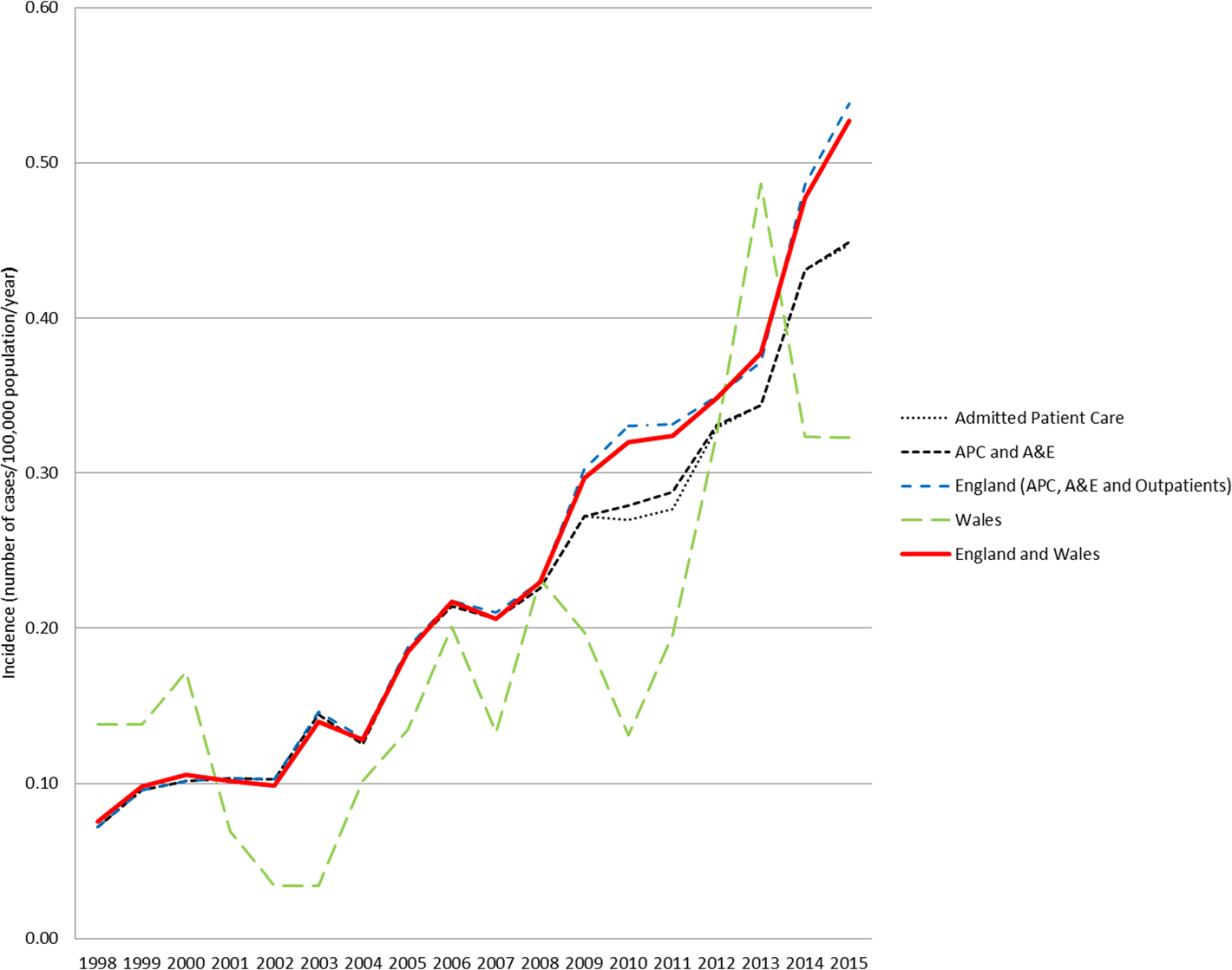
Incidence of Lyme disease coded patients within hospital administrative records in England and Wales (1998–2015)

**Fig. 2:**
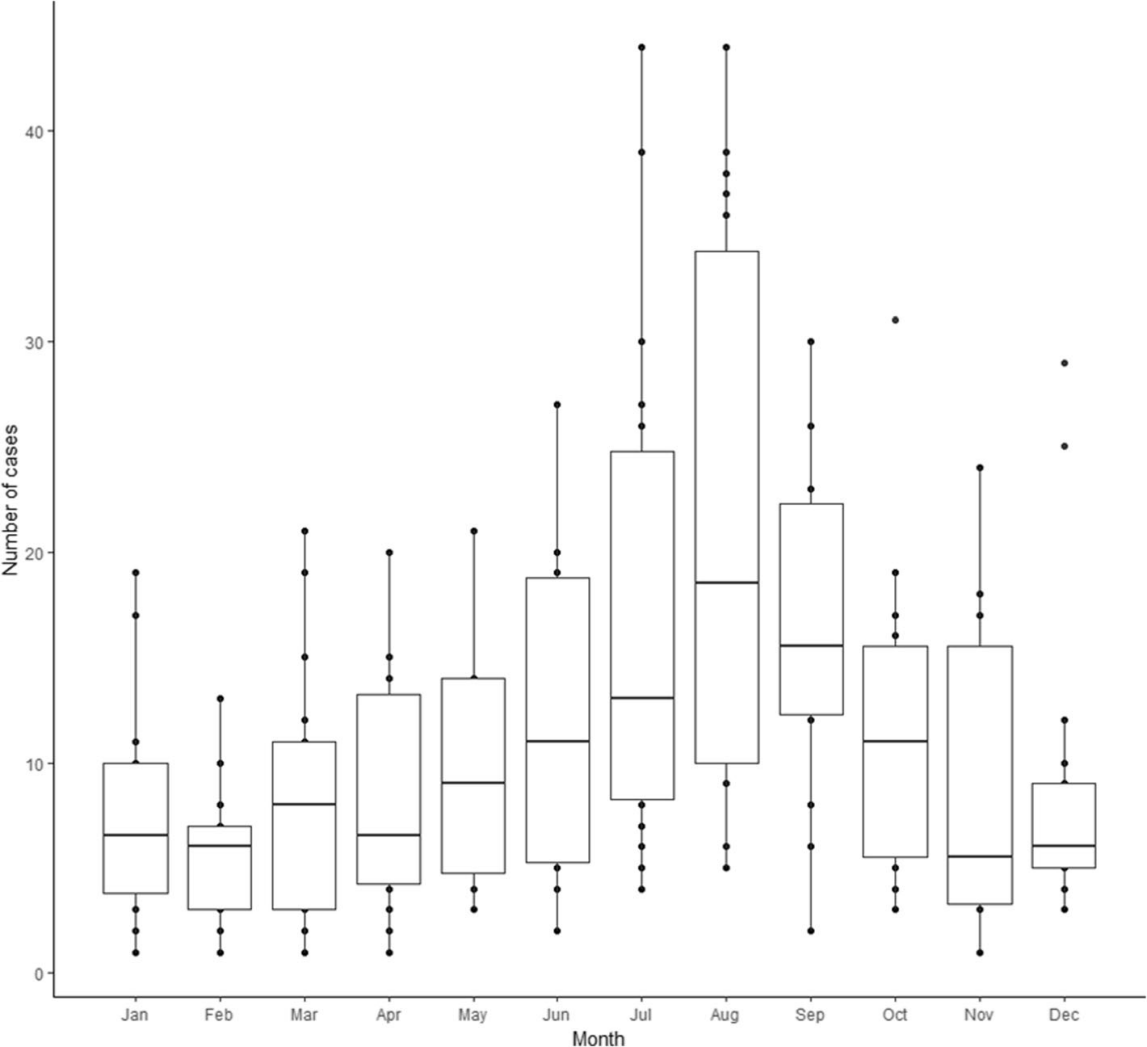
Lyme disease patient monthly count, within hospital administrative records in England and Wales (1998–2015)

The annual incidence of Lyme disease coded patients rose significantly from 0.08 cases per 100,000 population in 1998 to 0.53 in 2015 (r^2^ = 0.93, *p* < 0.01) (Fig. 1). This significant correlation was seen both in English (*r*^2^ = 0.93, *p* < 0.01) and Welsh (*r*^2^ = 0.55, *p* < 0.01) populations. There was marked seasonality, with peak number of cases recorded in August (Fig. 2).

### Demographic characteristics

**Fig. 3:**
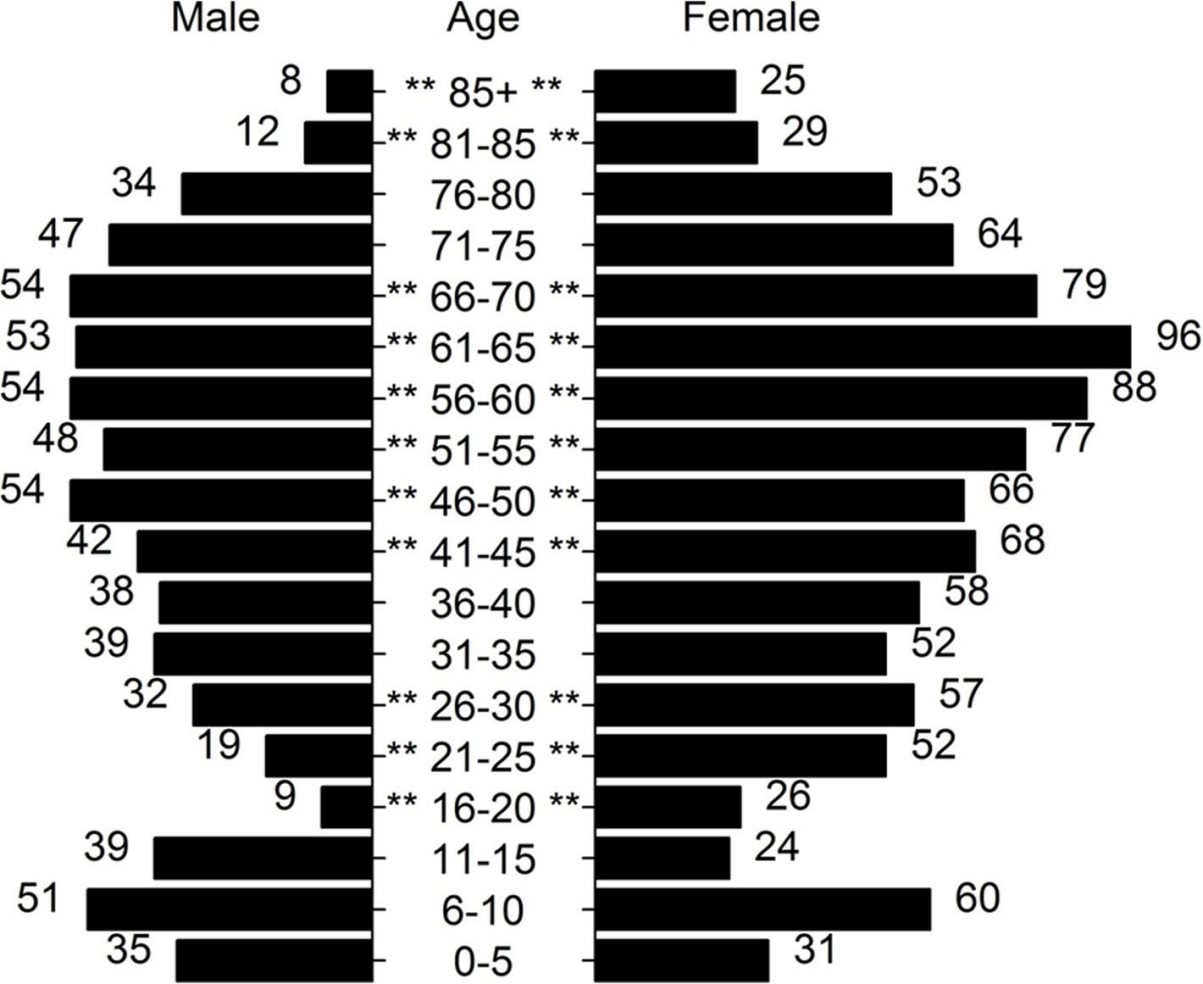
Population demographics of Lyme disease patients within hospital administrative records in England and Wales (1998–2015). Legend: Asterisks represent a significant difference (*p* < 0.05) between sexes

In England and Wales, 70.9% (*n* = 1673) of records contained information on the patients’ age and sex. When stratified by country, English records contained 69.5% (*n* = 1571) of this information, and Welsh 100% (*n* = 102). There were significantly (*p* < 0.01) more female patients than male in England and Wales 60.1% (*n* = 1005), displaying a bimodal age distribution, with peaks at 6–10 and 61–65 year age bands (Fig. 3). This sex ratio held true in England (60.5%, *p* < 0.01), in Wales there were more female patients 52.9% (*n* = 54), however this was not significant (*p* = 0.62). Ethnicity information was available for 79.5% (*n* = 1877) of records in England and Wales. Of these records, 96.1% (*n* = 1803) of patients were recorded as identifying with being white. Using a Chi-squared test to assess white ethnicity vs other ethnicities, a significantly (*p* = 0.01) greater proportion of this population was white compared to the 2011 Census population [22].

### Geographical distribution

**Fig. 4:**
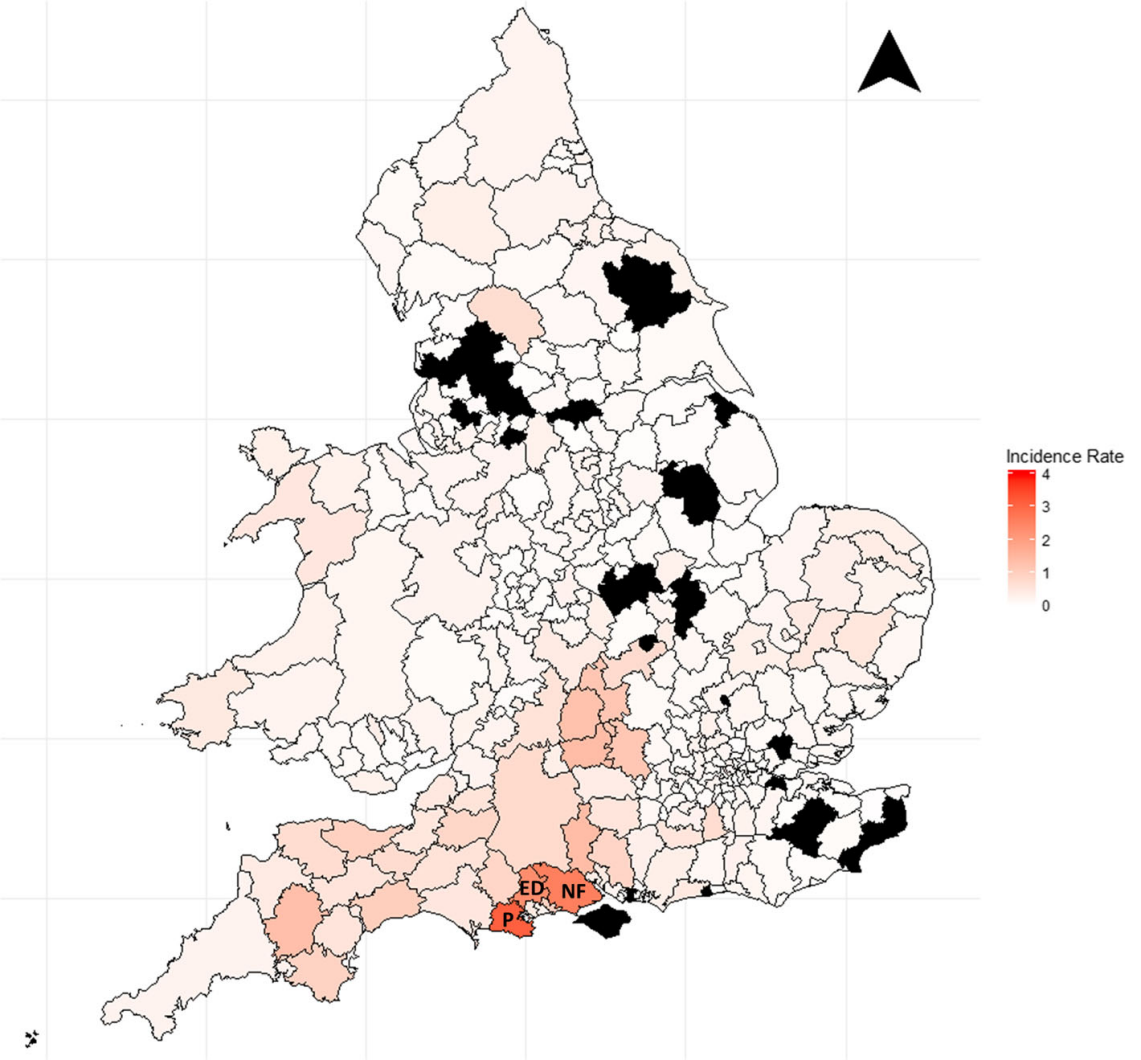
The average incidence rate of Lyme disease in English and Welsh local authorities (*n* = 348), (1998–2015). Legend: These data were based on hospital administrative records, and incidence measured as number of cases per 100,000 per year. Black areas recorded no cases over the study period. P = Purbeck, ED = East Dorset, NF = New Forest

Over two thousand (2078; 88.0%) records contained geographical information (Fig. 4). The areas with the highest incidence were located in south west of England. The local authorities with the highest incidence were Purbeck with 3.13 cases per 100,000 per year, New Forest (2.58), and East Dorset (2.32), with incidence in neighbouring areas in central southern England also with high rates. Thirty-four (9.8%) local authorities recorded no hospital cases assessed for Lyme disease.

Analysis of rural-urban indicators showed a significant difference between the study population (*n* = 2292) and the national population, where Lyme disease patients were more likely to live in rural rather than urban areas, compared to the national population (*p* < 0.01).

### Sociodemographic characteristics

Information on IMD deciles was available for 96.7% of English patients (*n* = 2186). There was a significant difference (*p* < 0.01) between this population and the national English population, with a significant linear trend showing that patients were found in increasing numbers in less deprived areas (*r*^2^ = 0.87, *p* < 0.01). Information on WIMD was available for 90.1% (*n* = 92) of Welsh patients; using Chi-squared tests, there was a significant difference (*p* < 0.01) between this population and the national Welsh population, and there was a significant linear trend, with increasing number of patients found in the least deprived areas (*p* < 0.01).

### Patient management

**Fig. 5:**
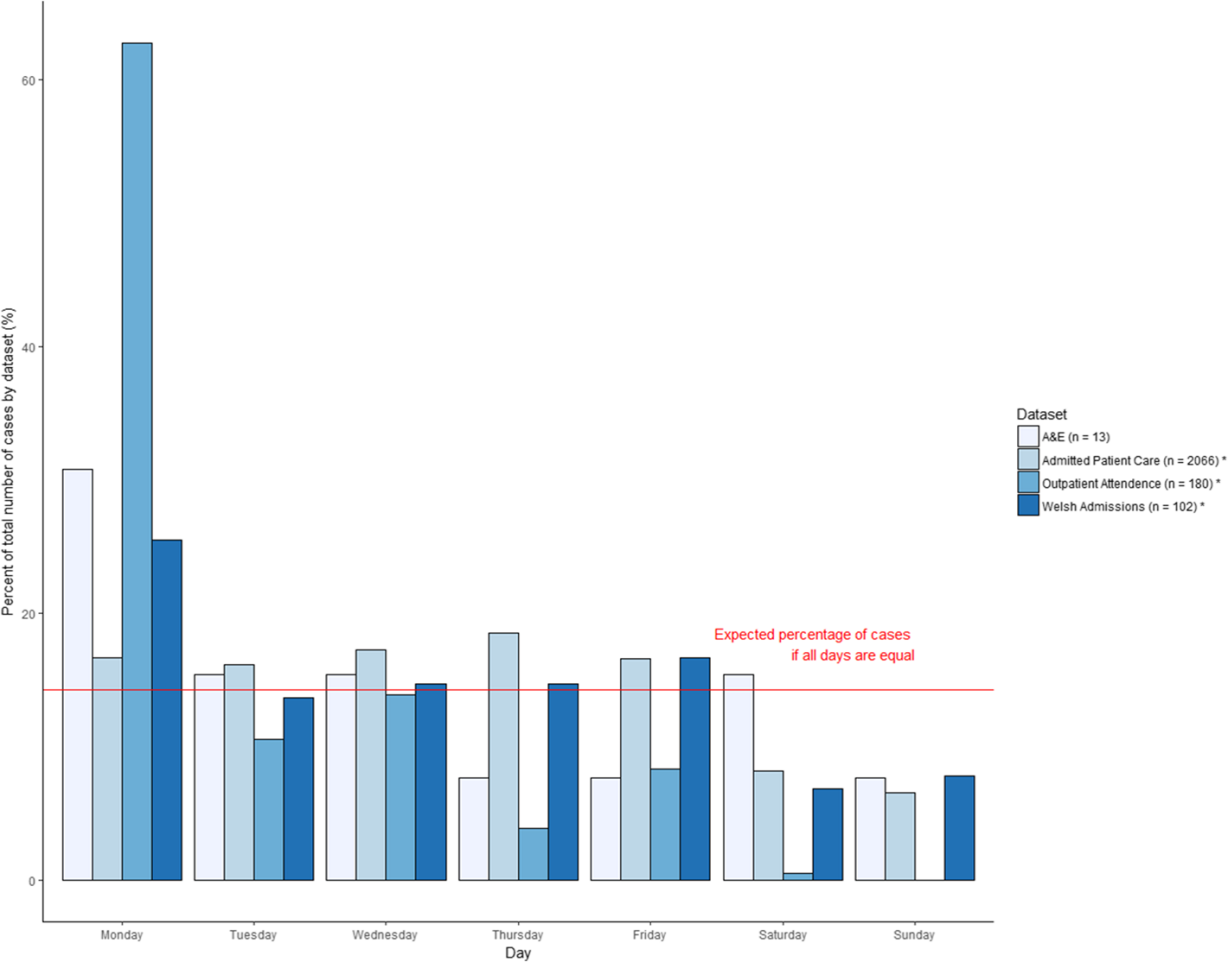
Proportional daily Lyme disease case attendance, in English and Welsh hospital administrative records (1998–2015). Legend: Asterisks represent a significant difference (*p* < 0.05) compared to the expected proportion of daily cases

**Fig. 6:**
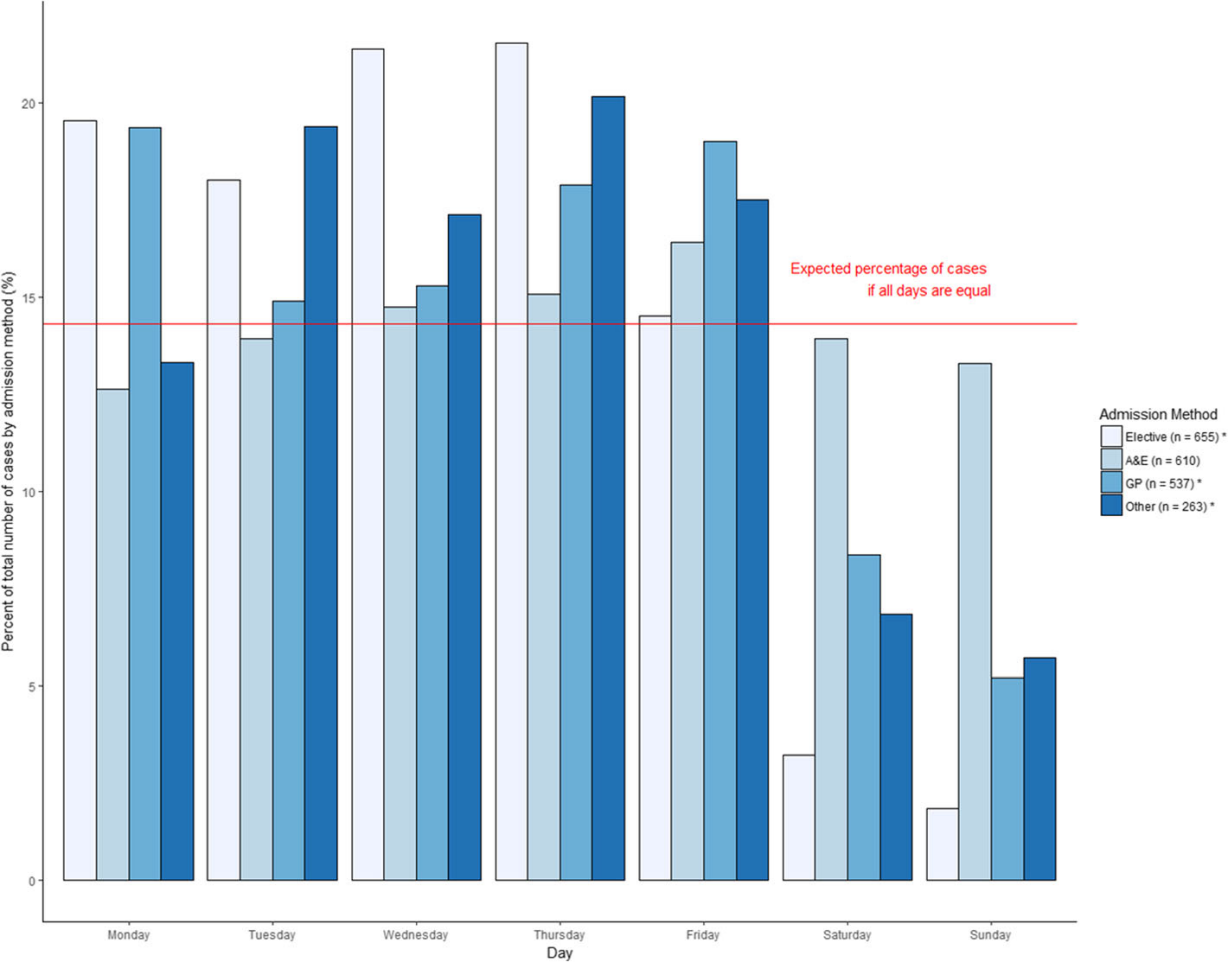
Proportional daily Lyme disease case admission routes, in English and Welsh hospitals (1998–2015). Legend: Asterisks represent a significant difference (*p* < 0.05) compared to the expected proportion of daily cases

There were significant differences between the daily cases in APC (*p* < 0.01), OA (*p* < 0.01), and Welsh admissions (*p* = 0.01), compared to the expected number of cases per day of the week. For these three datasets, there were fewer cases at the weekend, and the APC dataset had a high number of cases on a Monday (Fig. 5). There was no significant difference between daily case numbers for the A&E dataset (*p* = 0.72). Within the APC dataset, there were significant differences between the daily cases admitted via the elective (*p* < 0.01), GP (*p* < 0.01), and other (*p* < 0.01) routes, compared to the expected number of cases per day of the week. There were fewer cases admitted via these routes at the weekend. There was no significant difference between daily case numbers for patients admitted through A&E (*p* = 0.67) (Fig. 6).

In Table 3, coding patterns, department of treatment, bed days, number of appointments, and length of time in A&E for HES data, are shown.

**Table 3:** Patient management statistics for Lyme disease coded patients in Hospital Episode Statistics (1998–2015)

	Admitted Patient Care (APC)		Outpatients		Accident and Emergency (A&E)
ICD-10 Codes					
Lyme disease	91.5% (*n* = 1891)		27.8% (*n* = 55)		100% (n = 16)
Acrodermatitis chronica atrophicans	8.0% (*n* = 166)		71.4% (*n* = 142)		0
Lyme Arthritis	0.1% (*n* = 2)		0.5% (*n* = 1)		0
Lyme and LA	0.3% (*n* = 7)		0		0
Number of Departments of Treatment Recorded	63 (2065 patients)		20 (198 Patients)		N/A
Top 5 Departments of Treatment	General medicine	28.9%	Dermatology	70.7%	N/A
	Paediatrics	14.7%	Rheumatology	5.6%	
	Neurology	10.8%	Neurology	5.1%	
	Gynaecology	4.8%	Infectious disease	4.0%	
	Infectious disease	4.5%	General medicine	3.0%	
Mean number of episodes per patient	1.72 episodes (range: 1–50)		N/A		N/A
Mean number of bed days with patients with one episode (*n* = 1638, 79.3% of APC patients)	4.47 days (range: 0–137) 733 (35.5%) with one episode and no bed days. 258 (12.5%) with one episode and one bed day.		N/A		N/A
Mean number of total bed days for patients with more than one episode (*n* = 427, 20.7% of APC patients)	11.2 days (range: 0–315)		N/A		N/A
Mean number of outpatient appointments (*n* = 308, 24 cancelled)	N/A		1.5 (range: 1–25)		NA
Mean time in A&E (minutes)	N/A		N/A		140 (32–237)

Lyme disease was the predominant code in admissions (91.5%) and A&E (100%) data, where as it was ACA (71.4%) in outpatients. Data on patient management for PEDW data was limited to patient admission method; 67.6% (*n* = 69) of Welsh patients were admitted through the A&E department, the remainder were electively admitted.

**Fig. 7:**
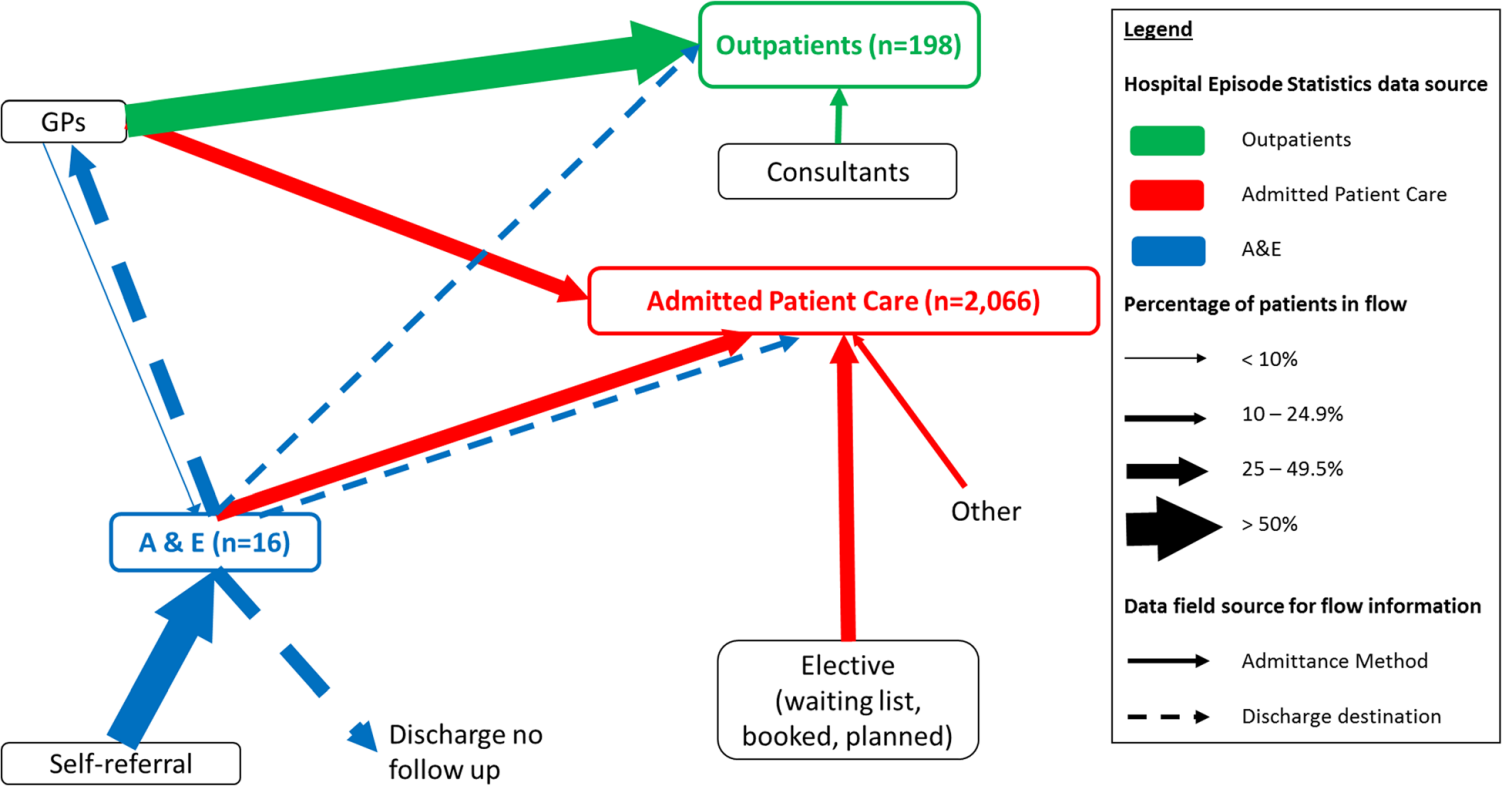
The pathway of Lyme disease coded patients through the NHS, based on hospital administrative records

Patient pathways were described using the source of the patient and their discharge method (Fig. 7). There was no discharge information for OA, and information for APC was excluded as discharge destination codes did not explicitly describe whether patients were to receive primary care on discharge or whether patients were referred to an outpatient or inpatient clinic.

## Discussion

This study provides an in-depth description of Lyme disease patients seen in English and Welsh hospitals, and addresses some of the NICE guidelines calls for new epidemiological data [5]. Incidence rose over the study period, showing a similar trend, but at lower levels, compared to officially published figures based on laboratory confirmed cases [2–4]. This discrepancy is to be expected, as national laboratories will receive samples from both hospital and primary care patients, and will therefore have a higher incidence. Not all cases would need to be referred to a hospital clinician from primary care, as the majority of cases are likely to present with an uncomplicated erythema migrans rash [5]. The cause for the increase in incidence is unknown, but may be the result of, among other causes; increased awareness by the public and/or hospital clinicians, increase in referrals by clinicians in primary care, or a true increase in incidence within England and Wales. Further research is needed to understand the drivers for this increase in incidence. Compared to other European countries the incidence we describe is lower. In France the annual hospitalisation rate due to Lyme diseases is 1.55 cases per 100,000 [23], with an estimated average national incidence of 42 cases per 100,000 population. Whereas in Germany the inpatient incidence was 9 cases per 100,000 population, but with large regional variation [24]. The reasons for this are mixed and are likely due to; differences in *Ixodes spp* prevalence and *Borrelia spp* carriage rates, different levels of exposure to ticks by the general population, and differences in how patients access healthcare.

The seasonality observed here supports the known risk factors and epidemiology of Lyme disease. Tick populations in the UK have been shown to peak in June or July each year [25–27]. One would therefore expect to see tick bite incidence and exposure to Lyme disease to peak similarly. Clinical signs will appear anywhere from several days to a few weeks after a tick bite [7]. Previous work in England and Wales showed a peak of serologically confirmed cases between July and September, with an assumed peak of symptoms earlier in the summer [4, 28]. This work would support this conclusion. This mirrors other Northern European countries, such as Finland and Germany, where clinically diagnosed cases peak throughout July and August [29, 30].

The age structure of this population compares closely with two recent studies performed in England and Wales [4, 15]. It shows the classic bimodal age distribution seen with Lyme disease, with an initial peak incidence in pre and peri-pubescent children, followed by a second larger peak from late middle age. The reasons for this age structure haven’t been formally assessed, however there is agreement that it likely reflects an increased exposure to tick habitats due to leisure behaviour rather than occupational exposure [30]. These data display a predominance of female cases, unlike both studies referenced above. The reasons for this are hard to explain, but could be related to differences in health seeking behaviour [31].

Ninety-six percent of patients identified as being white, compared to 86% in the 2011 national census [21]. There is no clear reason why ethnicity has any impact on a person’s susceptibility to Lyme disease. Instead, this apparent association is most likely due to sociocultural and behavioural reasons. Patients were found, in increasing numbers, living in less deprived areas. It must be noted that all ethnic minority groups were more likely to live in areas of higher deprivation than the white population [32], and this could explain the higher proportion of white patients within this population. Lyme disease patients were more likely than the national population to live in rural areas. The characterisation of Lyme disease patients as white and from suburban or rural areas with low deprivation may be explained by a complex combination of risk factors related to access to habitats which support ticks (either through work or recreation), and access to health care [33]. As deprivation and rural-urban data are derived from aggregated data, the exact location of a case’s Lyme disease acquisition and socioeconomic status is unknown. These data are therefore acting as proxies and it is unknown how representative they are of the individual case. Despite the identification of clear trends and associations, these factors cannot be unravelled using these datasets, and so the degree of inherent bias remains unknown. Further research, utilising multivariable models, is required to understand the link, and any interaction and confounding, between ethnicity, deprivation, area of residence and presentation to hospitals with Lyme disease.

There is clear geographical variation in incidence between local authorities. The highest incidence is in southern-central and western England, which has traditionally been seen as a Lyme disease hotspot [28]. Areas with no cases are unlikely to be due to an absence of disease but may reflect differences in case management or hospital coding practices. The remainder of England and Wales is a patchwork of low incidence with no obvious hotspots of disease. Interestingly, there are no clear foci of infection observed in either the Thetford Forest, the Lake District or the North Yorkshire Moors as identified previously by Public Health England (PHE) [2]. In these areas the awareness, diagnosis and management of Lyme disease may differ from other areas, perhaps with primary care clinicians treating cases in the community and with fewer subsequent cases referred to hospitals. This shows a similar geographic distribution to laboratory-confirmed cases of Lyme disease [4]. However, the areas of higher incidence in the hospital data expand further in to the south-west of England and into central England compared to laboratory cases. This is likely due to differences in case management. The high level of visual concordance between this research and the laboratory data suggest that both are accurately capturing the locations of Lyme disease patients. The geographical data collected by HES and PEDW is based upon the patient’s home address and no information is recorded on recent travel history or where a tick bite may have occurred, and so there may be an element of bias in the results. The data presented in this paper is at too low a geographical resolution and does not provide information on the patient’s tick bite location, to be able to hypothesise about any ecological associations with Lyme disease incidence.

Bed day analysis showed three distinct populations; those with one episode who weren’t admitted (35.5% of patients) or stayed for one night (12.5%), those with multiple episodes and a low number of bed days and those with one or many episodes that had a large number of bed days (Table 3). The first group is likely to represent patients with uncomplicated cases of Lyme disease. The second group often had consecutive daily episodes totalling 14 to 21 days, which could be consistent with daily intravenous doses of antibiotics as recommend by the British Infection Association and National Institute for Health and Care Excellence (NICE) guidelines [5, 8]. The final group appear to represent complicated cases of Lyme disease that require prolonged stays in hospital. It was not in the scope of this project to see whether any clinical presentations predisposed patients to these three groups, but further investigations are recommended.

Analysing the patient flow through the datasets has enabled better understanding of the care pathway for Lyme disease infected patients. Thirty percent of Lyme disease admissions in England, and 67.6% in Wales, originate from the A&E department. To place this into context, in 2011 69% of all NHS England admissions originate from A&E [34]. The same report saw a decline in admissions through primary care referral and an increase through A&E between 2001 and 2011. It would be unlikely that the numbers of patients admitted in our study have more acute/severe presentations of disease that require immediate hospital attendance, however this cannot be ruled out. A combination of two factors possibly result in this finding; the lack of knowledge of the recommended care pathways for symptoms associated with Lyme disease (such as flu-like illness and rashes), and the difficulty in getting a prompt appointment in primary care [12, 34–37]. Peak non-urgent attendance at NHS emergency departments has been recorded at weekends [38], which may be due to the lack of access to primary care at the weekend [12, 35–37, 39]. However, our data show that the number of cases appearing in A&E is relatively evenly distributed throughout week, suggesting that the lack of knowledge of where to seek help with Lyme disease symptoms may be the predominant cause of the above findings. Further work is needed to explore why so many patients would seek treatment at a hospital when, for the majority of cases, management could occur at primary care level. By linking with primary care electronic health records, one may be able to see whether they had sought help first in primary care before arriving at A&E.

The major limitations of this study revolve around the use and validity of ICD-10 codes. A case of Lyme disease can be defined without laboratory confirmation, so there is no way to independently validate the accuracy of diagnostic coding in this context [5, 8]. Previous work has shown that coding practices in hospitals are not infallible, but are steadily improving; quality issues were primarily focused on patient management variables, rather than demographics and geography [40]. Without such an audit, any potential inconsistencies in coding behaviour cannot be fully understood or quantified. Subjectively, admissions data in HES and PEDW were the most robust. As such, further work on the Lyme disease patient hospital population should primarily focus on admissions data.

Sixty-three treatment departments were recorded, some of which have no discernible link to Lyme disease. This may represent simple coding errors or that the code has been added for completeness when the primary reason for admission was unrelated to Lyme disease. The outpatient dataset was significantly overrepresented by two hospitals; both had the main treating department as dermatology and resulted in a high number of ACA codes. This is further seen, by the large number of outpatients seen on a Monday. These cases were all from one hospital, and likely represent one dermatology clinician’s outpatient clinic. This suggests that outpatient departments across England and Wales were not coding consistently and episodes may be being lost. The A&E dataset contained very low numbers of patients, in stark contrast to the large number being admitted through A&E as recorded in the APC dataset. The main reasons for these low numbers is not through lack of attendance but how coding is encouraged. Within A&E, coding is not required to be as specific as the admissions data, and is just needed to code a generalised condition, sub-analysis of more serious conditions and anatomical area involved [41]. This results in Lyme disease potentially falling into multiple categories depending on symptoms, such as “Infectious disease”, “Local infection”, “Dermatological conditions” and “Facio-maxillary conditions”. This has been seen in previous work on arthropod bites, where all cases were recorded as “Bites/Stings” and routinely didn’t specify the causal arthropod [42].

PEDW only collects admission data and so some of the issues discussed above for the English dataset were negated. Unfortunately linkage between the PEDW and HES datasets was not possible; though, for reasons described above, these patients were likely to be unique. Without linkage there still is the potential of duplication of patients within the records and therefore there is a small degree of uncertainty attached to these results.

## Conclusions

This study has, for the first time, described the demographics of hospital patients who are coded with Lyme disease, across England and Wales. The demography of this population poses some interesting questions, especially around female predominance, the relative lack of ethnic diversity and the trend towards habitation in areas of low deprivation. This study provides a platform to inform future work on Lyme disease patients within hospital settings. Analysis of secondary care data can inform and help target health promotion messages, and as this is an ongoing dataset, interventions relating to Lyme disease could be formally assessed.

## Data Availability

The data governance arrangements for the study do not allow us to redistribute HES data to other parties. Researchers interested in accessing HES data can apply for access through NHS Digital’s Data Access Request Service (DARS) https://dataaccessrequest.hscic.gov.uk/. The data governance arrangements for the study do not allow us to redistribute PEDW data to other parties. Researchers interested in accessing PEDW data can apply for access through the NHS Informatics Service – PEDW Data Online http://www.infoandstats.wales.nhs.uk/page.cfm?orgid=869&pid=40977

## Abbreviations

A&E: Accident and emergency
ACA: Acrodermatitis chronica atrophicans
APC: Admitted patient care
HES: Hospital Episode Statistics
ICD-10: International Statistical Classification of Diseases and Related Health Problems 10th Revision
IMD: Index of Multiple Deprivation
LSOA: Lower super output area
NHS: National Health Service
NICE: National Institute for Health and Care Excellence
OA: Outpatient attendance
ONS: Office for National Statistics
PEDW: Patient Episode Database for Wales
PHE: Public Health England
WIMD: Welsh Index of Multiple Deprivation
